# Editorial: New insights into gut microbiota in colorectal cancer

**DOI:** 10.3389/fcimb.2026.1788000

**Published:** 2026-02-04

**Authors:** Neeraj Kapur, Syed Adeel Hassan

**Affiliations:** 1 VA Lexington Medical Center, Lexington, KY, United States; 2 Department of Internal Medicine-GI, University of Kentucky College of Medicine, Lexington, KY, United States; 3 Department of Gastroenterology and Hepatology, University of Michigan Michigan Medicine, Ann Arbor, MI, United States

**Keywords:** colorectal cancer, dysbiosis, immune, microbiome, neoadjuvant

Colorectal cancer (CRC) arises from complex interactions among genetic, lifestyle, and environmental factors and remains the second leading cause of cancer-related mortality. Despite advances in screening, immunotherapy, and neoadjuvant treatments, mortality rates remain high, underscoring the need for diversified therapeutic strategies. The significant heterogeneity in treatment response further highlights gaps in comparative and mechanistic understanding. Recent evidence implicates the host–tumor–microbiome axis as a critical modulator of disease progression, emphasizing the importance of integrating clinical outcome–driven research with emerging therapeutic frameworks.

This Research Topic integrates evidence from four original research investigations and four cutting edge review manuscripts that collectively highlight the emerging convergence between therapeutic strategies, tumor biology and the gut microbiome in colorectal (CRC). Together, these contributions integrate real−world clinical evidence with conceptual advances that advance the framework for personalized and precision management of CRC.

Zhang et al. presented a three-arm retrospective study comparing conventional chemoradiotherapy, total neoadjuvant therapy (TNT), and PD-1–based immunotherapy in locally advanced rectal cancer (LARC). Their findings indicate that incorporating TNT or immunotherapy into the neoadjuvant regimen enhances pathological response and tumor downstaging, bolstering evidence for a paradigm shift toward intensified multimodal therapy. Yet, the study also exposes key diagnostic limitations, including inconsistent MRI restaging accuracy and restricted microbiome sampling, which hinder robust biomarker validation. The authors emphasize the need for multicenter, prospective investigations combining radiomics and microbiome profiling to advance precision in treatment response prediction.

Extending this biological continuum, Wang et al. delineated profound gut microbial dysbiosis in CRC, marked by enrichment of pro-tumorigenic taxa Fusobacterium and Peptostreptococcus. The study’s integrative analysis connects these microbial profiles with oncogenic gene expression signatures (TIMP1, BCAT1, TRPM4, MYBL2), suggesting the role of a coordinated microbiota–gene network shaping CRC progression. Although limited by sample size and cross-sectional design, these results strengthen the growing recognition of the gut microbiome as a biomarker reservoir and a modifiable therapeutic target in colorectal carcinogenesis. However, the absence of functional and metabolomic validation constrains mechanistic insight, underscoring the need for larger longitudinal multi−omics studies to establish causality and therapeutic relevance. Similarly, Kushwaha et al. performed a culture-based study in Indian CRC patients wherein potentially pro-tumorigenic genera were enriched including Enterococcus, Escherichia, Klebsiella, and Veillonella compared with adjacent normal mucosa. Isolation of 75 viable strains from paired sites underscores the value of cultivable bacteria as experimentally tractable candidates to interrogate inflammation, carcinogenesis and develop a specific microbial profile. Findings here reinforce the utility of gut microbiome as a reservoir of putative diagnostic markers and targets for microbiota-modulating interventions. However, the small cohort size, single-center setting, and exclusive reliance on culture constrain generalizability and likely underestimate microbial diversity. Larger, longitudinal studies integrating culture with sequencing-based profiling are needed to distinguish microbial drivers from passengers in colorectal cancer.

Complementing these findings from the upper GI tract, Qian et al. applied a multi-omics approach to Gastric Cancer (GC), revealing that microbiome-associated epigenetic dysregulation may underlie gastric tumorigenesis. Six key hub genes (CDK1, CDK2, NOXO1, CUL1, MAPK1, CCNB1) emerged as consistently altered across transcriptomic and methylation datasets, with functional validation in GC cell lines. This work broadens the molecular framework linking microbial influence on gene regulation in GC and highlights novel candidates for biomarker and drug development. However, as with related studies, the lack of direct patient microbiome data and mechanistic exploration underscores the current gap between computational inference and biological validation.

Taken together, these investigations illuminate the dynamic interplay between external microbial ecosystems and internal tumor epigenetics, with therapeutic implications spanning the GI axis. As immunotherapy continues to be incorporated into earlier disease settings and microbiome research matures into interventional science, an integrated systems approach will be essential. Future research must bridge clinical oncology with microbiome and epigenetic biology through harmonized, longitudinal, multi-omics trials. The convergence of these domains heralds a new era of precision GI oncology, one that not only targets the tumor but also the microbial and molecular landscapes that shape its evolution.

The accompanying review articles converge on a unifying message. The gut microbiome is a central, actionable driver of colorectal cancer biology, simultaneously shaping tumor initiation, progression and therapeutic response through tightly interwoven immune, metabolic and barrier mechanisms. Dysbiosis, marked by loss of butyrate-producing commensals and overgrowth of pathobionts such as Fusobacterium nucleatum, enterotoxigenic Bacteroides fragilis and toxigenic E. coli, emerge as both initiator and amplifier of malignant evolution thereby linking diet, sex hormones and host genetics to DNA damage, epigenetic rewiring, chronic inflammation and an immunosuppressive niche. In parallel, commensal-derived short-chain fatty acids and selected probiotics exert opposing, tumor-constraining effects, while the same microbial circuits that govern carcinogenesis also gatekeep efficacy and toxicity of chemotherapy, radiotherapy and immune checkpoint blockade. This aids in the positioning of microbiome-informed biomarkers and microbiota-targeted interventions (including probiotics and fecal microbiota transplantation) as integral components of future precision prevention and therapy in CRC.

Viewed as a whole, clinical response in CRC is jointly determined by therapeutic regimen, intrinsic tumor features, host immunity and the gut microbiota. Current response assessment tools remain poorly calibrated to this complexity. The integration of outcome-oriented clinical studies with mechanistic reviews heightens translational relevance and provides a roadmap for biological insight driven regimen selection, sequencing and de-escalation. Several priorities emerge: prospective comparative trials of TNT and immunotherapy-based strategies integrated into coordinated multi-omics platforms; improved imaging, molecular and microbial predictors of response and toxicity; and deeper characterization of microbiome–immune–tumor interactions across the disease continuum, including survivorship. By elevating the utility of microbiome to the same level as tumor genomics and host factors, the evidence supports a future in which GI oncology targets not only cancer cells, but also the microbial and molecular ecosystems that drive their parallel evolution.

In summary, this Research Topic provides a timely and cohesive overview of emerging neoadjuvant and biologically informed approaches in colorectal and rectal cancer. We hope this Research Topic stimulates further investigation and guides clinicians and researchers toward more precise and effective treatment paradigms.

